# Sinapic acid modulates oxidative stress and metabolic disturbances to attenuate ovarian fibrosis in letrozole‐induced polycystic ovary syndrome SD rats

**DOI:** 10.1002/fsn3.3973

**Published:** 2024-01-24

**Authors:** Huan Lan, Zhe‐Wen Dong, Ming‐Yu Zhang, Wan‐Ying Li, Chao‐Jie Chong, Ya‐Qi Wu, Zi‐Xian Wang, Jun‐Yang Liu, Zhi‐Qiang Liu, Xiao‐Hui Qin, Tie‐Min Jiang, Jia‐Le Song

**Affiliations:** ^1^ Guangxi Key Laboratory of Environmental Exposureomics and Entire Lifecycle Health Guilin Medical University Guilin Guangxi China; ^2^ College of Chinese Material Medica Guangzhou University of Chinese Medicine Guangzhou Guangzhou China; ^3^ College of Pharmacy Shenyang Pharmaceutical University Shenyang Liaoning China; ^4^ South Asia Branch of National Engineering Center of Dairy for Maternal and Child Health Guilin University of Technology Guilin Guangxi China; ^5^ Department of Obstetrics and Clinical Nutrition The Second Affiliated Hospital of Guilin Medical University Guilin Guangxi China; ^6^ Guangxi Key Laboratory of Health Care Food Science and Technology Hezhou University Hezhou Guangxi China

**Keywords:** fibrosis, metabolic disturbances, oxidative stress, polycystic ovary syndrome, sinapic acid, TGF‐β1/Smads

## Abstract

Sinapic acid (SA) is renowned for its many pharmacological activities as a polyphenolic compound. The cause of polycystic ovary syndrome (PCOS), a commonly encountered array of metabolic and hormonal abnormalities in females, has yet to be determined. The present experiment was performed to evaluate the antifibrotic properties of SA in rats with letrozole‐induced PCOS‐related ovarian fibrosis. SA treatment successfully mitigated the changes induced by letrozole in body weight (BW) (*p* < .01) and relative ovary weight (*p* < .05). Histological observation revealed that SA reduced the number of atretic and cystic follicles (AFs) and (CFs) (*p* < .01), as well as ovarian fibrosis, in PCOS rats. Additionally, SA treatment impacted the serum levels of sex hormones in PCOS rats. Luteinizing hormone (LH) and testosterone (T) levels were decreased (*p* < .01, *p* < .05), and follicle‐stimulating hormone (FSH) levels were increased (*p* < .05). SA administration also decreased triglyceride (TG) (*p* < .01) and total cholesterol (TC) levels (*p* < .05) and increased high‐density lipoprotein cholesterol (HDL‐C) levels (*p* < .01), thereby alleviating letrozole‐induced metabolic dysfunction in PCOS rats. Furthermore, SA treatment targeted insulin resistance (IR) and increased the messenger RNA (mRNA) levels of antioxidant enzymes in the ovaries of PCOS rats. Finally, SA treatment enhanced the activity of peroxisome proliferator‐activated receptor‐γ (PPAR‐γ), reduced the activation of transforming growth factor‐β1 (TGF‐β1)/Smads, and decreased collagen I, α‐smooth muscle actin (α‐SMA), and connective tissue growth factor (CTGF) levels in the ovaries of PCOS rats. These observations suggest that SA significantly ameliorates metabolic dysfunction and oxidative stress and ultimately reduces ovarian fibrosis in rats with letrozole‐induced PCOS.

## Graphical abstract

Sinapic acid (SA) has the ability to deactivate the transforming growth factor‐β1 (TGF‐β1)/Smad signaling pathway and enhance the expression of peroxisome proliferator‐activated receptor‐γ (PPAR‐γ). This suggests that SA might have potential therapeutic benefits in preventing fibrosis in the ovary.

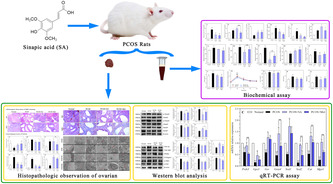

## 1 INTRODUCTION

Polycystic ovary syndrome (PCOS) is a prevalent disorder affecting a significant proportion of reproductive‐age women, typically between 4 and 21%. The condition is characterized by hyperandrogenism, polycystic ovaries, and either infrequent or absent ovulation, and it primarily manifests as an endocrine and metabolic imbalance (Xie et al., 2019). The pathogenesis of PCOS has been suggested to be multifactorial, involving the hypothalamic–pituitary–ovarian (HPO) axis dysfunction, obesity, insulin resistance (IR), oxidative stress, ovarian fibrosis, lipid imbalance, and genetic defects (Anagnostis et al., 2018; Bhati et al., 2020).

The existing evidence thus far indicates that women with PCOS frequently demonstrate ovarian fibrosis, which is intrinsic to infertility and is considered one of the primary features (Kakoly et al., 2019). Oxidative stress‐induced ovarian damage can trigger ovarian fibrosis (Khaje Roshanaee et al., 2022), consequently contributing to physiological disturbances such as menstrual abnormalities and reproductive disturbances (Ainehchi et al., 2020; Shokoohi et al., 2022; Zhou et al., 2017). Recent research has suggested that women with PCOS and ovulatory dysfunction may have an increase in the production of extracellular matrix (ECM), which can lead to interstitial fibrosis, a thickening of the capsule, and a thickening of the basement membrane (Zych et al., 2019). Individuals diagnosed with PCOS have high levels of transforming growth factor‐β1 (TGF‐β1) in their blood (Takahashi et al., 2017). TGF‐β1 is a multifunctional cytokine that activates Smad proteins and promotes fibrosis in many kinds of organs and tissues (Yang et al., 2021). Activation of transforming growth factor beta receptor 1 (TGF‐βR1) leads to the phosphorylation of Smad2/3, which are subsequently joined with Smad4 and translocated to the nucleus (Wang, Li, et al., 2020). Furthermore, it has been widely accepted that peroxisome proliferator‐activated receptor‐γ (PPAR‐γ) influences the inflammatory response in the ovaries of PCOS patients (Prabhu & Valsala Gopalakrishnan, 2020). Studies have indicated that PPAR‐γ has a powerful antifibrotic action, impacting myofibroblasts, restraining the expression of fibrosis factors, and obstructing the activation of TGF‐β1/Smads (Prabhu & Valsala Gopalakrishnan, 2020). Blockade of TGF‐β1/Smads’ activation can effectively improve tissue and organ fibrosis (Györfi et al., 2018).

Treatment of PCOS patients using conventional insulin‐sensitizing medications has been linked to a variety of adverse effects, including gastrointestinal problems, lactic acidosis, and renal insufficiency (Lashen, 2010). It is therefore vital that new alternative therapies for the treatment of PCOS with minimal side effects are available and under investigation. Plant‐derived phenolic compounds have been extensively utilized to ameliorate female reproductive and metabolic issues due to their capacity to scavenge free radicals and provide chemoprotection (Jahan et al., 2018). Sinapic acid (SA) is a well‐known polyphenol found in several herbs (such as borage, sage, mace, and rosemary), fruits (such as strawberries, lemons, blueberries, kiwi, plums, cherries, and apples), cereals (oats), and some vegetables (such as kale, broccoli, and turnips) in the Brassicaceae family (Bin Jardan et al., 2020; Zare et al., 2015). Some pharmacological investigations have reported that SA has antioxidant, antianxiety, anticolitis, anticancer cell growth, intestinal barrier maintenance, anti‐inflammatory, immunomodulatory, antifibrotic (liver and lung), antihypertensive, and antimicrobial activities and improves lipid metabolism (Jabbar et al., 2023; Lan et al., 2021; Li et al., 2023; Qian et al., 2020; Wang et al., 2022; Zhao et al., 2021). This study intends to assess SA's impact on ovarian fibrosis in rats with PCOS, as the efficacy of SA in preventing ovarian fibrosis has not yet been explored.

## 2 MATERIALS AND METHODS

### 2.1 Chemical reagents

Metformin (Met, purity >98%), SA (CAS No.: 530‐59‐6, purity >98%), carboxymethyl cellulose (CMC), and letrozole were received from Topscience Co. Ltd. (Shanghai, China). Goat antirabbit immunoglobulin G (IgG) and other primary antibodies (Smad2, connective tissue growth factor (CTGF), p‐Smad2, TGF‐β1, Smad7, Smad3, and p‐Smad3) were received from Beyotime Biotech Inc (Shanghai, China). Papanicolaou staining solution and primary antibodies (collagen I, PPAR‐γ, β‐actin, α‐smooth muscle actin (α‐SMA), Smad4, and TGF‐βR1) were provided by Servicebio Technology Co., Ltd. (Wuhan, China).

### 2.2 In vivo experimental design and animal grouping

Hunan SJA Experimental Animal Co., Ltd. (Changsha, China) supplied six‐week‐old Sprague–Dawley (SD) rats (female, 190–210 g). All animals were fed AIN‐93G diets and provided water in a sun protection factor (SPF) environment (23°C ± 1°C, 12 h of light and 12 h of darkness). After adaptation, all animals were randomly divided intofour groups (eight rats/group) as follows: the normal rats were gavaged with CMC (1%); the PCOS rats were gavaged with letrozole (1 mg/kg) suspension; the SA‐treated PCOS rats were gavaged with letrozole (1 mg/kg) + SA (50 mg/kg); and the Met‐treated PCOS rats were gavaged with letrozole (1 mg/kg) + Met (265 mg/kg). SA and Met were both dissolved in 1% CMC, and the experiment was conducted for a total of 21 days (Zhou et al., 2021). The investigation method of this study was approved by the Institutional Animal Care and Use Committee (IACUC) of Guilin Medical University (review code: GLMC‐201806003).

### 2.3 Estrus cycle detection for a papanicolaou staining assay

The detection of the estrous cycle was performed using a protocol reported in our previous studies (Chen, 2016; Shin et al., 2013). Vaginal smears were performed on rats for eight consecutive days starting on the 10th day of dosing. A swab soaked with saline was placed clockwise into the rat's vagina and then applied to a slide and stained with Papanicolaou stain to observe changes in the estrous cycle by microscopy.

### 2.4 Blood collection and oral glucose tolerance test (OGTT)

All animals were fasted for 12 h prior to being anesthetized with Zoletil 50 (50 mg/kg; Virbac Laboratories, Carros, France), after which blood samples were obtained for the fasting blood glucose (FBG), OGTT, and insulin resistance (IR) assays. Glucose (2 g/kg BW) was administered orally using a gastric tube, and blood glucose was subsequently assessed after 0, 30, 60, 90, and 120 min using a kit (Beyotime) to determine the levels of FGB and glucose load. Glucose tolerance is expressed as a function of the area under the OGTT curve (area under the curve (AUC)) and the postload glucose responses (Adeyanju et al., 2020). Additional blood samples (5 mL) were collected from the inferior vena cava into vacuum blood collection tubes (Solarbio) and centrifuged (3000 × *g*, 10 min at 4°C) using a D1524R high‐speed refrigerated centrifuge (DLAB Scientific Co., Ltd, Beijing, China) to separate the serum and determine the IR levels (Guerrero‐Romero et al., 2010).

### 2.5 Determination of sex hormones and lipid concentrations in serum

The serum concentrations of sex hormones (i.e., rat luteinizing hormone (LH), estradiol (E2), follicle‐stimulating hormone (FSH), and testosterone (T)) were detected by colorimetry kits (Jiangsu Meimian Industrial Co. Ltd., Jiangsu, China). The serum levels of lipid profiles, superoxide dismutase (SOD), and malondialdehyde (MDA) were determined by the corresponding colorimetry kits provided by Beyotime.

### 2.6 Histopathologic observation of the ovaries

The ovaries and periuterine fat were fixed with a reagent (4% paraformaldehyde (PFA)) for 24 h and embedded in paraffin. All ovarian sections (5‐μm thick) were stained with hematoxylin and eosin (H&E) and Masson trichrome staining solution (Servicebio) and observed using an Olympus CX31 microscope (Olympus Co., Tokyo, Japan). For each group, the numbers of corpora lutea (CLs) and primary, secondary, antral follicles, atretic follicles (ATFs), and cystic follicles (CFs) per ovary were counted. The primary follicle was identified by the iso‐ to highly prismatic structure of the follicular epithelium that enveloped the oocyte. The presence of a secondary follicle was indicated by small fluid‐filled cavities among the granulosum cells. The classification of an antral follicle (AF) was determined by the presence of a follicle with two or more layers of cuboidal granulosa cells (GCs) and the visibility of the cavity. AF included follicles containing degenerating ova or pyknotic GCs. Follicles with large fluid‐filled structures and attenuated granulosa cell layers (GCLs), along with thickened theca interna cell layers, were accepted as CFs.

Following fixation of the other ovarian portions in 5% glutaraldehyde, a routine method, which included 1% osmium tetroxide, was used to prepare them for electron microscopic examination. The ultrathin sections were cut to a thickness of 50 nm with a Reichert Ultracut S ultramicrotome and then double‐stained with lead citrate and uranyl acetate before being observed using an HT7700 transmission electron microscope (Hitachi High‐Technologies Corporation, Japan).

### 2.7 Collecting total RNA and quantitative real‐time (qRT) PCR assay

The messenger RNA (mRNA) levels of the target genes were assessed with a qRT‐PCR Kit (T2210; Solarbio, China) using a QuantStudio 6 Flex Real‐Time PCR System (Thermo Fisher Scientific, Waltham, MA, USA). The relative gene expression level was calculated according to the 2^–ΔΔCt^ method described under the same conditions in our previous report (Zhou et al., 2021).

### 2.8 Western blot analysis

One ovary (approximately 40 mg) was ground in a KZ‐II High‐throughput tissue grinder (Servicebio), after which the total protein was extracted. The well‐boiled protein samples were separated by 10% or 12% BeyoGel™ Plus Precast Tris‐Gly PAGE Gel (Beyotime). Then, they were blocked with QuickBlock™ buffer (Beyotime) for 15 min, incubated with primary antibodies (collagen I, p‐Smad2, Smad2, CTGF, TGF‐β1, p‐Smad3, Smad3, PPAR‐γ, β‐actin, Smad4, α‐SMA, and Smad7) for 3.5 h at 4°C, and then incubated with antirabbit IgG for 90 min at 37°C. Subsequently, all bands were observed in the FluorChem M System (ProteinSimple, Santa Clara, CA, USA) after reaction with the Enhanced Chemiluminescence (ECL) detection kit, and bands were analyzed for grayscale using ImageJ (https://imagej.nih.gov/ij/, NIH Image, Bethesda, MD, USA).

### 2.9 Immunohistochemical (IHC) staining assay

The previously processed ovarian sections were dewaxed, rehydrated, sealed with hydrogen peroxide (H_2_O_2_) (3%) to block endogenous peroxidase for 30 min, placed in ethylenediaminetetraacetic acid (EDTA) repair solution for antigen repair for 30 min, and then blocked using 10% goat serum for 15 min before being incubated with TGF‐β1, Smad2, Smad3, Smad4, and Smad7 primary antibodies. Then, all sections were washed and incubated with secondary antibody. They were then incubated with horseradish peroxidase (HRP)‐–streptavidin (Beyotime) at 37°C for 20 min. All sections were incubated with 3,3′‐diaminobenzidine (DAB) solution, rinsed in tap water, and observed under a microscope. A brown color represented a positive reaction.

### 2.10 Statistical analysis

The investigation included repeating all experiments three times, and the data are presented as the mean ± standard deviation (SD). One‐factor analysis of variance (ANOVA) and Bonferroni's test were used to determine statistically significant differences among the groups (*p* < .05) using SPSS 22.0 software (SPSS Inc., Chicago, USA).

## 3 RESULTS

### 3.1 Effect of SA on irregular estrous cycles in PCOS rats

**FIGURE 1:**
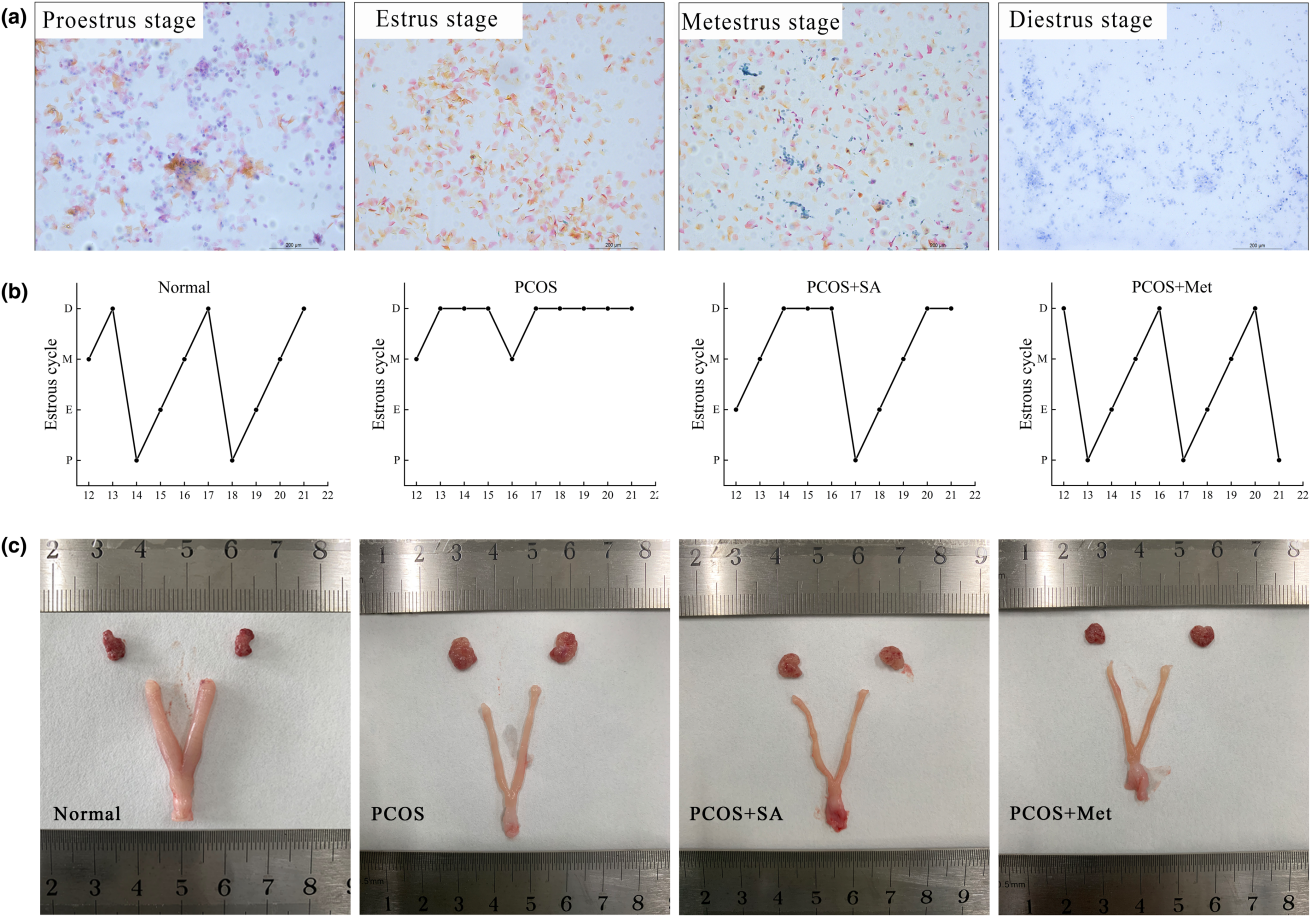
Microscopic characteristics of (a) the estrous cycle in the control rats and (b) dynamic changes in the estrous cycle in the four groups. (c) The changes in vaginal exfoliative cytology in rats were determined by Papanicolaou staining assays (scale bar = 200 μm). The appearances of rat ovaries and uteri are shown.

The PCOS model was evaluated by analyzing changes in the sexual cycle. As exhibited in the pictures from the normal rats, the proestrus smear was predominantly composed of nuclear epithelial cells, the estrus smear was chiefly made up of cornified cells, the metestrus smear was an equal mix of leukocytes, epithelial cells, and cornified cells, and the diestrus smear was mostly composed of leukocytes (Figure 1a). All four stages, proestrus, estrus, metestrus, and diestrus, appeared to be regular in the normal group (Figure 1b). The vaginal smear of the PCOS group showed continuous estrus or diestrus stages, indicating a prolonged estrus cycle and confirming the successful modeling of PCOS in rats. However, both SA and Met restored the estrous cycle in PCOS rats when compared to the PCOS rats without treatment.

### 3.2 Morphological observations of the ovaries and uterus in SA‐treated PCOS rats

The PCOS group exhibited generally pale ovarian and uterine tissues (Figure 1c). Conversely, the ovaries and uterus in the normal rats exhibited a plentiful blood supply and a deep red color. The PCOS + SA and PCOS + Met groups experienced a positive change in the pathological lesions.

### 3.3 Histological structure of the ovaries in SA‐treated PCOS rats

**FIGURE 2:**
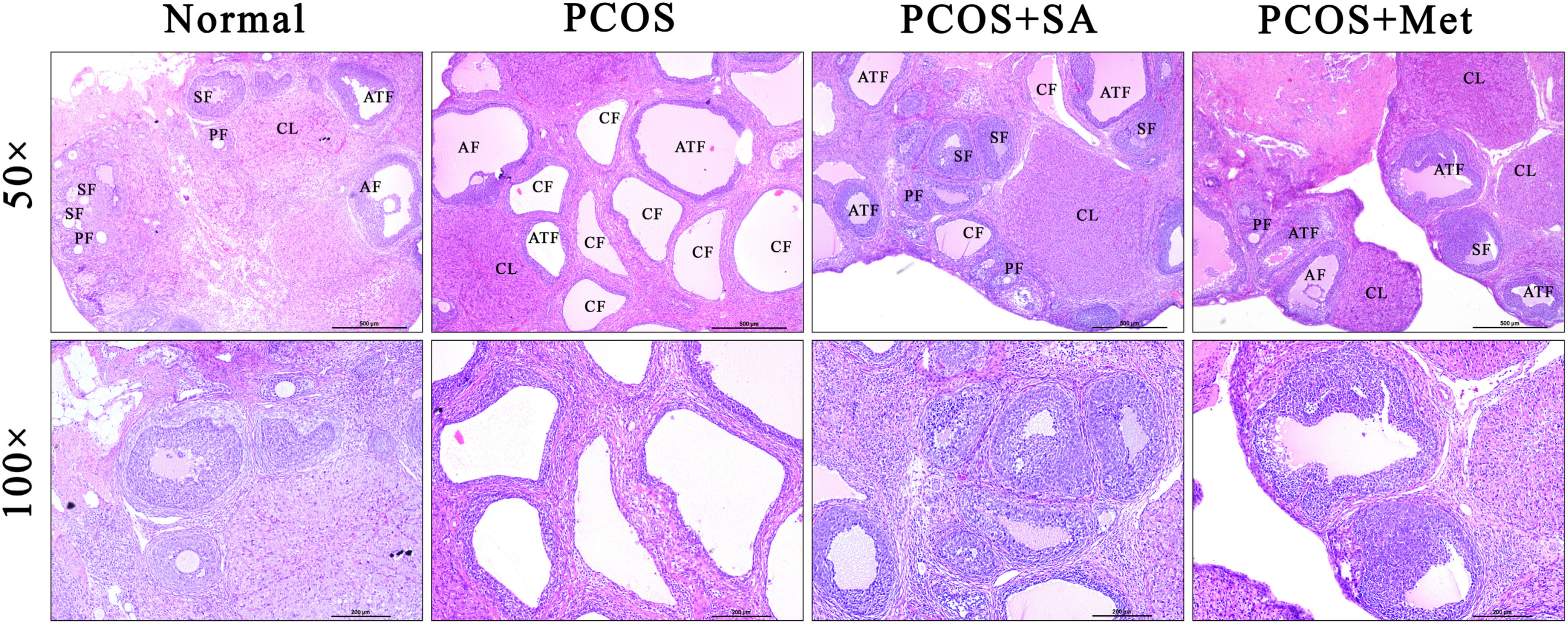
Histological observations in the ovaries of SA‐treated rats (scale bar = 500 μm, high‐magnification scale bar = 200 μm).

Figure 2 shows that the sections from the normal rats displayed normal follicles at different developmental stages and CLs, which indicate ovulation. The arrangement of granulosa and theca cells (TCs) was orderly and complete. In contrast, the PCOS group exhibited cystic expansion, significantly enlarged cystic sinus follicles, thinned GCLs, and dramatically reduced numbers of CLs, which are consistent with the pathological changes in the ovaries of PCOS patients. However, the ovarian structure in PCOS rats receiving SA and Met treatments returned to levels similar to those of the normal rats. The GCLs became thicker and regularly arranged, and there were many CLs and significantly reduced numbers of cystic follicles. Additionally, Table 1 shows that there were significantly fewer primary follicles in the PCOS rats than in the normal rats (*p* < .01). Notably, the primary follicle count in the SA‐treated PCOS rats was significantly higher than that in the PCOS rats (*p* < .05). Similarly, Met‐treated PCOS rats had a higher count of primary follicles than the PCOS rats (*p* < .01). There were no significant differences in the counts of secondary follicles among all animals. The count of antral follicles (AFCs) was higher in the PCOS rats than in the normal rats (*p* < .01). There was no significant difference in AFC between the PCOS + SA group and the PCOS group, but the AFC was significantly lower in the Met‐treated group than in the other PCOS group (*p* < .05). The numbers of AF and CF were significantly increased in the PCOS group compared to those in the normal group (*p* < .01), and these numbers were significantly decreased in the SA‐ and Met‐treated PCOS rats (*p* < .01). The CL count was significantly lower in PCOS rats than in normal rats (*p* < .01), but the CL counts were markedly higher in the SA and Met treatment groups than in the PCOS group (*p* < .01).

**TABLE 1:** Effect of SA on development levels of follicles in the ovaries of PCOS rats.

	Control	PCOS	PCOS + SA50	PCOS + SA150
PF	7.40 ± 2.79	2.80 ± 1.09**	5.8 ± 0.84#	7.00 ± 2.24##
SF	3.25 ± 1.26	4.50 ± 0.58	3.50 ± 0.58	3.50 ± 1.29
AF	1.50 ± 0.58	3.75 ± 0.50**	3.75 ± 1.26	2.50 ± 0.58#◆
ATF	1.75 ± 0.50	6.25 ± 0.96**	2.50 ± 0.58##	3.00 ± 0.82##
CF	0.25 ± 0.50	9.75 ± 0.96**	2.50 ± 0.58##	2.00 ± 0.82##
CL	6.00 ± 0.82	2.00 ± 0.82**	8.00 ± 0.82##	6.75 ± 0.50##

*Note*: *p < .05, **p < .01 vs. the normal group; #*p* < .05, ##*p* < .01 vs. the PCOS group; ♦*p* < .05, ♦♦*p* < .01, the PCOS + SA group vs. the PCOS + Met group. The data are shown as the mean ± SD.

Abbreviations: AF, antral follicle; ATF, atretic follicle; CF, cystic follicle; CL, corpus luteum; PF, primary follicle; SF, secondary follicle.

### 3.4 SA attenuated fibrosis in the ovaries of PCOS rats

**FIGURE 3:**
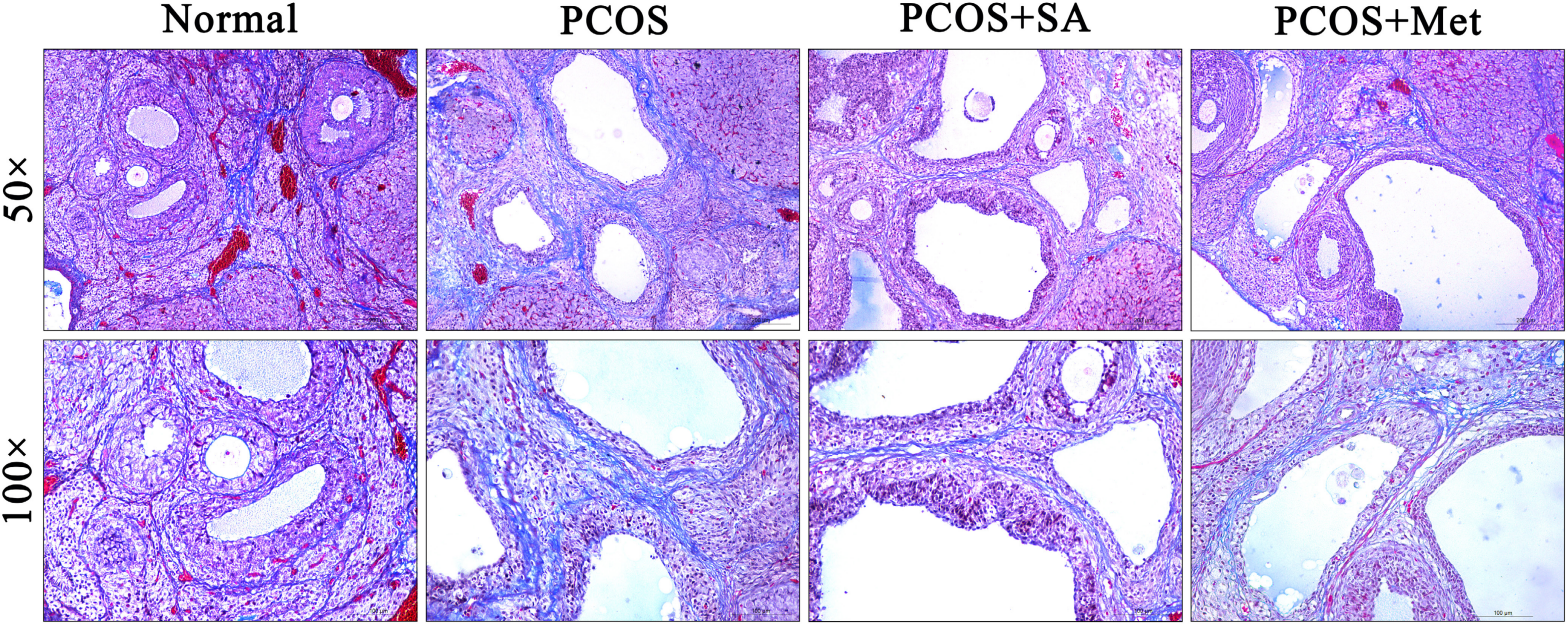
Masson's staining observations of the ovaries of PCOS rats treated with SA.

Ovarian sections from PCOS rats exhibited dramatically increased levels of fibrosis compared with those from normal rats (Figure 3). Fibrosis generation was significantly lower in both SA‐ and Met‐treated rats than in PCOS rats. SA treatment showed a better ability to reduce fibrosis than Met treatment in PCOS rats.

### 3.5 Transmission electron microscopy (TEM) observation of the ovaries in SA‐treated PCOS rats

**FIGURE 4:**
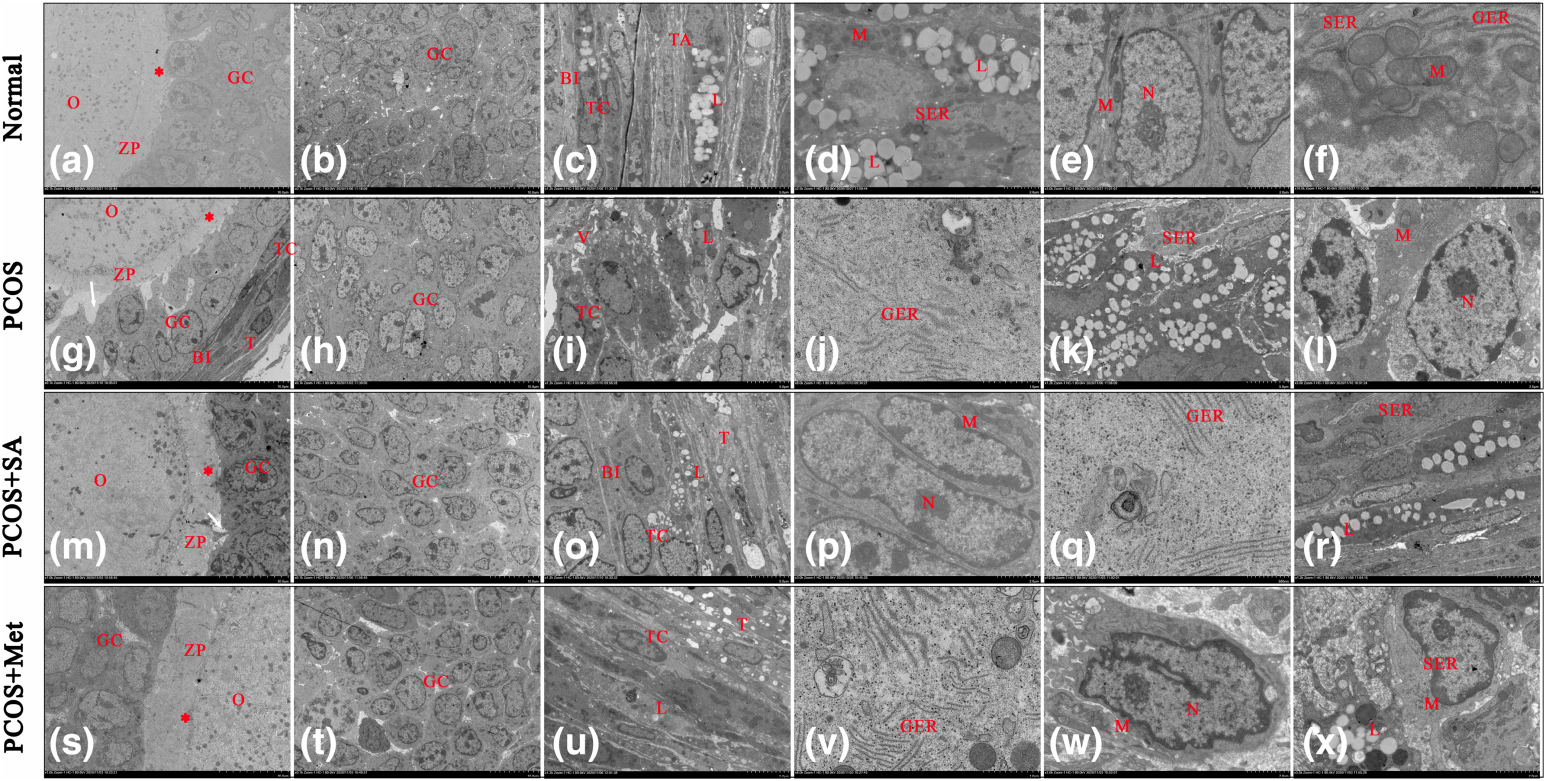
Electron micrographs of ovaries (a–x). Arrow: large spaces, *, microvilli; BI, basal lamina; GC, granulosa cell; GER, granular endoplasmic reticulum cisternae; L, lipid droplets; M, mitochondria; N, nucleus; O, oocyte; SER, smooth endoplasmic reticulum cisternae; TC, theca cell; ZP, zona pellucida. Bar = 2 μm.

When normal rats were examined, the oolemmas surrounding the oocytes were uniform and integrated with the adjacent follicular cells (FCs), and scattered mitochondria with intact cristae were observed in the cytoplasm of the oocytes (Figure 4a). Oocytes with nuclei and mitochondria in the cytoplasm, along with corpus luteum cells containing lipid droplets, a well‐developed smooth endoplasmic reticulum (SER), a granular endoplasmic reticulum (GER), and mitochondria were also observed (Figure 4d,f). Mitochondria and a uniform membrane structure were observed in the GCs (Figure 4b,e). TCs in the follicles exhibited typical characteristics of steroid‐expressing cells and contained lipid droplets (Figure 4c). All cells, GER, SER, and mitochondria appeared to have normal morphology. The PCOS group exhibited a considerable number of degenerative changes in the follicles. An abundance of apoptotic GCs spilled into the antrum, and a thickened zona pellucida (ZP) and shrunken GCs were present (Figure 4g). Some follicles showed expanded intercellular spaces between GCs (Figure 4h). Similarly, there were large spaces between the thickened ZP and inner GCs (Figure 4g). The GCLs of CF were only 1–2 cells in height and were surrounded externally by a thickened theca layer (Figure 4i). The GCs exhibited enlargement of SER and GER, as well as clumping of heterochromatin in the nucleus. Swollen and degenerated mitochondria were observed in the cytoplasm (Figure 4j,k). An increase in the intercellular space, degenerated mitochondria with deteriorated cristae structures, and excessive lipid accumulation were found in the theca cell layer (TCL) (Figure 4i,l).

Upon examination of oocytes from the PCOS + SA and PCOS + Met groups, an intact ZP structure and integrity between the surrounding GCs were observed (Figure 4m,n,s,t). The FCs of the PCOS + SA and PCOS + Met groups contained fewer degenerated mitochondria, lipid droplets, and apoptotic GCs than those in the PCOS group (Figure 4o,u). Healthy mitochondria with cristae structures were detected in the GCs (Figure 4r,w). It was also observed that the structures of the nucleus, GER, and SER in these cells had returned to normal morphology (Figure 4r,v). Regular arrangements of the TCL and a healthy mitochondrial structure were also observed in these cells (Figure 4p,x). In the PCOS + SA group, there were some spaces between the thickened ZP and the inner GCs (Figure 4m).

### 3.6 Effects of SA on BW and visceral adipose weight in PCOS rats

**FIGURE 5:**
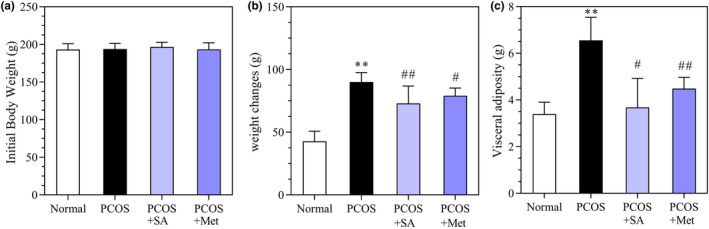
Effects of SA on body weight (BW) and visceral adiposity in rats with letrozole‐induced PCOS. Initial BW (a), final BW (b), and visceral adiposity (c). *p < .05, **p < .01 vs. the normal group; #*p* < .05, ##*p* < .01 vs. the PCOS group; ♦*p* < .05, ♦♦*p* < .01, the PCOS + SA group vs. the PCOS + Met group. The data are shown as the mean ± SD.

Figure 5 shows that the BW and visceral adipose weight were significantly higher in PCOS rats than in normal rats (*p* < .01). The BW and visceral adipose weight of both the SA and Met treatment groups were lower than those of the PCOS group (*p* < .01 and *p* < .05, respectively).

### 3.7 Effects of SA on the serum levels of FBG, AUC of OGTT, fasting insulin (FINS), and homeostatic model assessment for insulin resistance (HOMA‐IR) in PCOS rats

**FIGURE 6:**
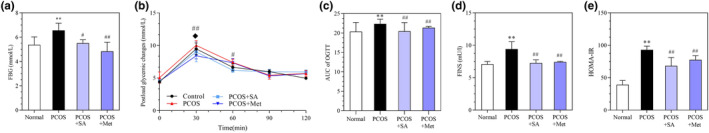
Effects of SA on (a) the blood levels of FBG and (b) the oral glucose tolerance test, (c) AUC of OGTT, (d) FINS, and (e) HOMA‐IR results in rats with letrozole‐induced PCOS. *p < .05, **p < .01 vs. the normal group; #*p* < .05, ##*p* < .01 vs. the PCOS group; ♦*p* < .05, the PCOS + SA group vs. the PCOS + Met group. The data are shown as the mean ± SD.

Both the SA and Met treatments reduced the serum FBG values compared to those of the untreated PCOS rats (*p* < .05 and *p* < .01, respectively) (Figure 6). The AUC values for OGTT, FINS, and HOMA‐IR were significantly higher for the PCOS rats than for the normal rats (*p* < .01). In contrast, these AUC values were significantly lower in both the SA‐ and the Met‐treated groups than in the PCOS group (*p* < .01).

### 3.8 Effects of SA on the serum levels of sex hormones in PCOS rats

**FIGURE 7:**
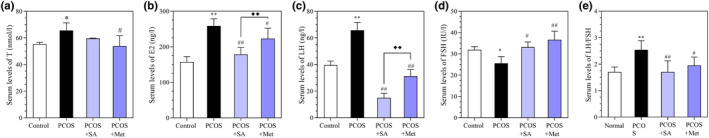
Effects of SA on (a) T, (b) E2, (c) LH, and (d) FSH levels and (e) the LH/FSH ratio in rats with letrozole‐induced PCOS. *p < .05, **p < .01 vs. the normal group; #*p* < .05, ##*p* < .01 vs. the PCOS group; ♦♦*p* < .01, the PCOS + SA group vs. the PCOS + Met group. The data are shown as the mean ± SD.

The serum T levels were higher in the PCOS group than in the normal group (*p* < .05), and the T levels were significantly lower in the PCOS + Met group than in the PCOS group (*p* < .05) (Figure 7). A significant reduction in the LH/FSH ratios was observed in the SA and Met treatments in comparison to the PCOS rats (*p* < .01 and *p* < .05, respectively). It was observed that the levels of LH and E2 in SA‐ and Met‐treated PCOS rats were significantly lower than in untreated PCOS rats, and that the serum levels of E2 and LH in SA‐treated PCOS rats were more markedly reduced than in Met‐treated PCOS rats (*p* < .01). The serum FSH levels were lower in the PCOS rats than in the normal rats (*p* < .05), and both SA and Met treatments increased the FSH levels to levels higher than those of the PCOS rats (*p* < .05 and *p* < .01, respectively).

### 3.9 Effects of SA on the serum levels of sex hormones in PCOS rats

**FIGURE 8:**
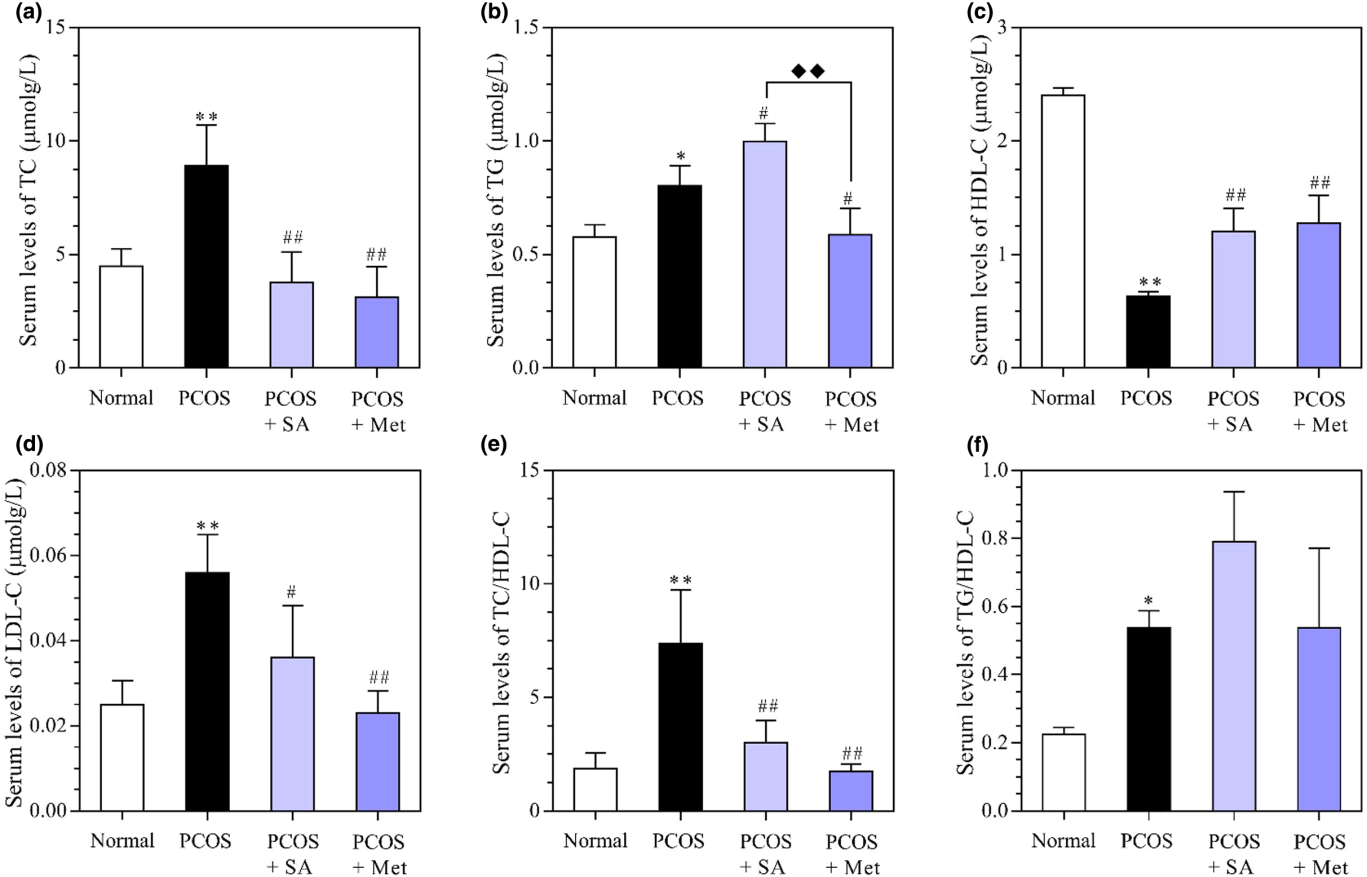
Effects of SA on (a) TC, (b) TGs, (c) HDL‐C, (d) LDL‐C, (e) the TG/HDL‐C ratio and (f) the TC/HDL‐C ratio in rats with letrozole‐induced PCOS. *p < .05, **p < .01 vs. the normal group; #*p* < .05, ##*p* < .01 vs. the PCOS group; ♦*p* < .05, ♦♦*p* < .01, the PCOS + SA group vs. the PCOS + Met group. The data are shown as the mean ± SD.

In both the SA‐ and Met‐treated PCOS rats, the serum levels of TC were lower than those in the PCOS group (*p* < .01) (Figure 8). Additionally, the levels of triglyceride (TG) and total cholesterol/high‐density lipoprotein cholesterol (TC/HDL‐C) were higher in the PCOS + SA group than in the PCOS group (*p* < .05), and the levels of these markers were lower in the PCOS + Met group than in the PCOS group (*p* < .01). Furthermore, the levels of TG were lower in the Met‐treated PCOS group than in the SA‐treated group (*p* < .01). In both the SA‐ and Met‐treated PCOS groups, the serum levels of HDL‐C were higher than those in the nontreated PCOS group (*p* < .01). Interestingly, the levels of LDL‐C were significantly lower in the PCOS + SA and PCOS + Met groups than in the PCOS group (*p* < .05 and *p* < .01, respectively).

### 3.10 Effects of SA on the serum levels of SOD and MDA in PCOS rats

**FIGURE 9:**
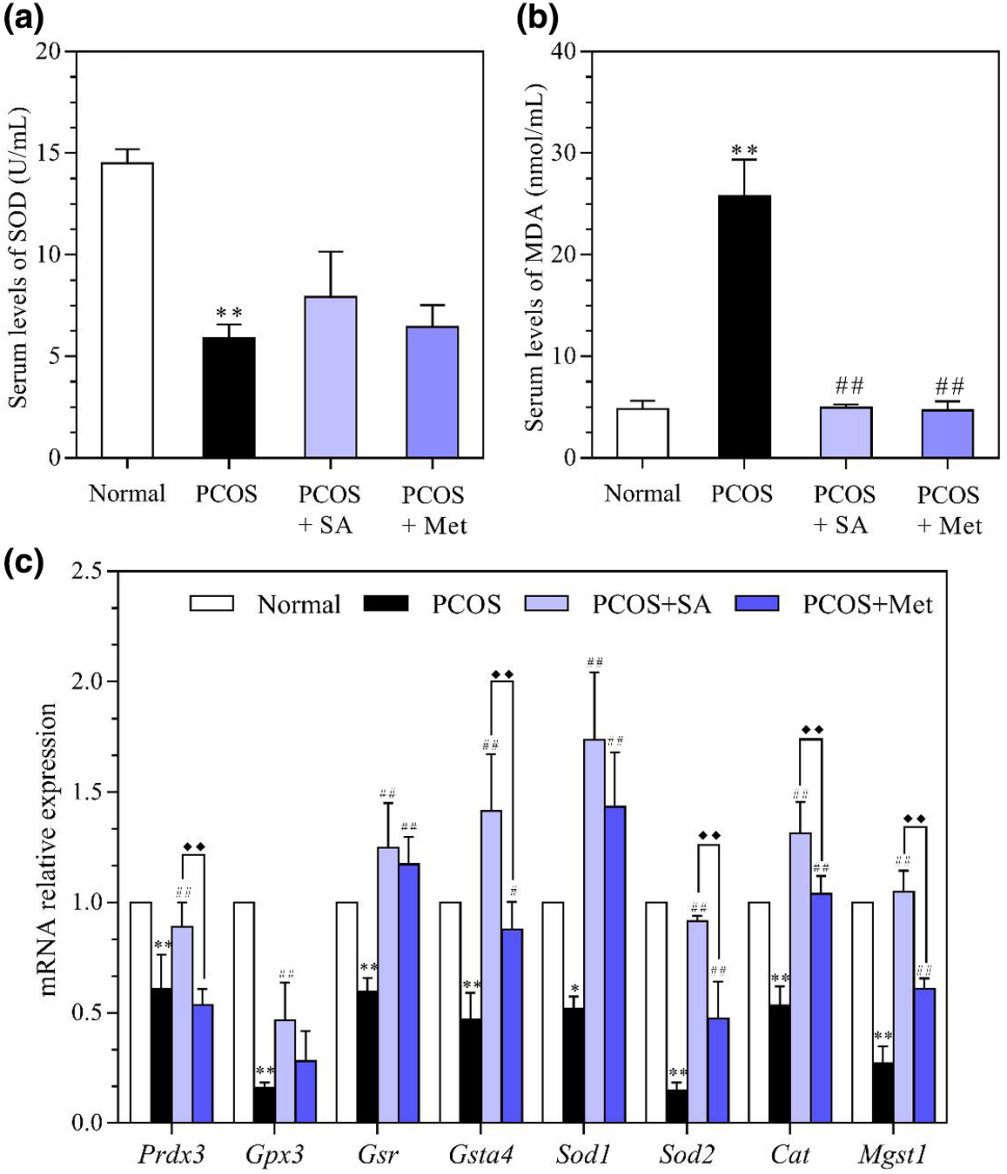
Effects of SA on the levels of SOD (a) and MDA (b) and the mRNA levels of antioxidant enzymes (c) in rats with letrozole‐induced PCOS. Ovarian mRNA expression of Cat, Sod2, Gpx3, Mgst1, Gsta4, Gsr, Sod1, and Prdx3 was analyzed by quantitative RT–PCR assays. β‐Actin was used as an internal control, and the relative mRNA level of each gene was normalized to the mRNA level in the normal group. *p < .05, **p < .01 vs. the normal group; #*p* < .05, ##*p* < .01 vs. the PCOS group; ♦*p* < .05, ♦♦*p* < .01, the PCOS + SA group vs. the PCOS + Met group. The data are shown as the mean ± SD.

Figure 9 reveals a significant decrease in serum levels of SOD in the PCOS rats (*p* < .01). No notable discrepancies in serum levels of SOD were observed among all PCOS groups, which included both the SA and Met treatments. Conversely, the serum levels of MDA were substantially increased in the PCOS group (*p* < .01). Administration of SA and Met was able to reduce the serum levels of MDA in the PCOS rats compared to the untreated PCOS rats (both *p* < .01).

### 3.11 Effects of SA on the mRNA levels of antioxidant factors in the ovarian tissues of PCOS rats

As shown in Figure 9c, SA administration significantly increased the levels of *Prdx3*, *Gsta4*, *Gpx3*, *Gsr*, *Sod1*, *Sod2*, *Cat*, and *Mgst1* compared with those in the PCOS rats (*p* < .01). Similarly, Met administration increased the levels of *Gsr*, *Sod1*, *Sod2*, *Cat*, and *Mgst1* compared to those in the PCOS rats (*p* < .01). The *Gsta4* levels in the Met‐treated PCOS rats were significantly higher than those in the PCOS rats (*p* < .05). The increase in the mRNA levels of *Gsta4*, *Sod2*, *Cat*, and *Mgst1* was much more pronounced in Met‐treated PCOS rats than in SA‐treated PCOS rats (*p* < .01).

### 3.12 Effects of SA on the protein levels of Smads in the ovarian tissues of PCOS rats

**FIGURE 10:**
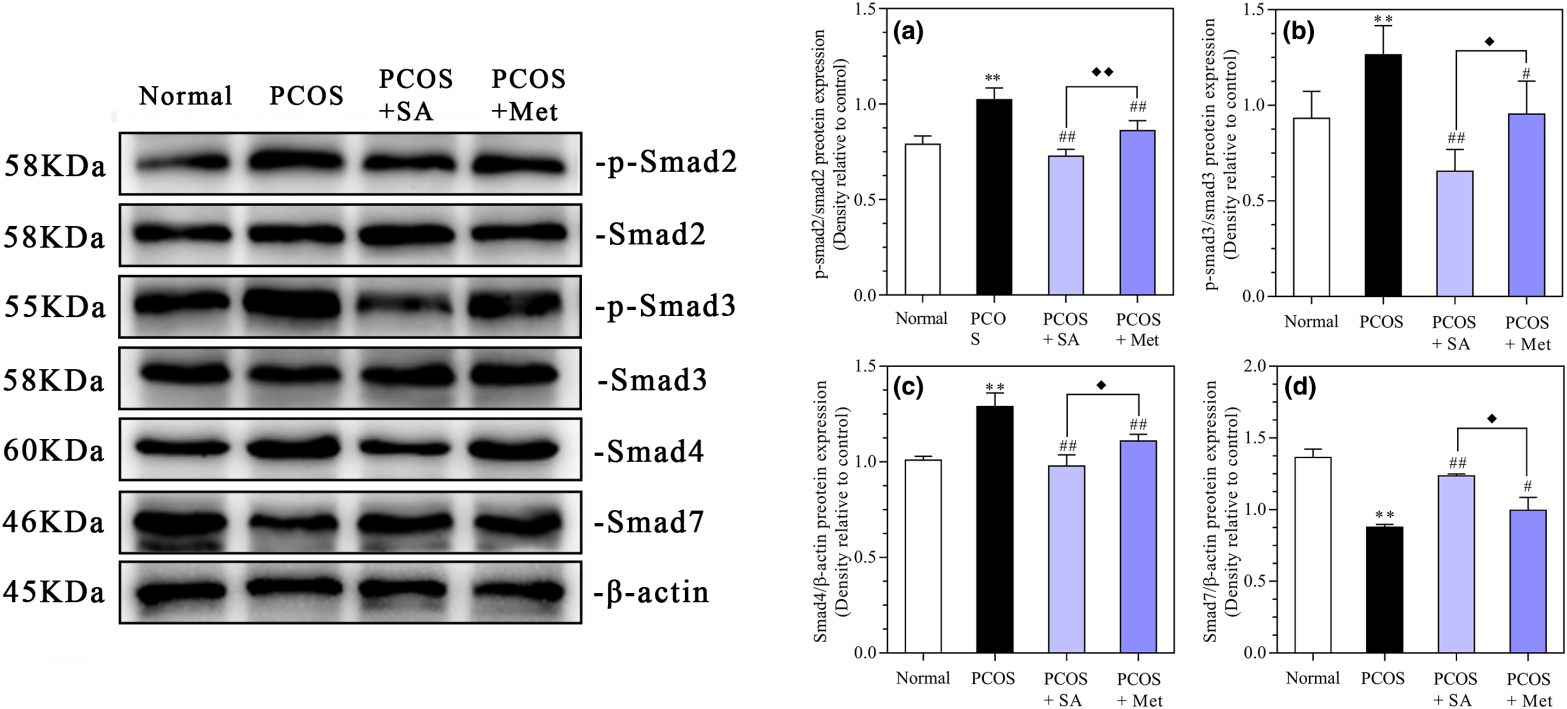
Effects of SA on the protein levels of p‐Smad2/Smad2 (a), p‐Smad3/Smad3 (b), Smad4 (c), and Smad7 (d) in rats with letrozole‐induced PCOS. *p < .05, **p < .01 vs. the normal group; #*p* < .05, ##*p* < .01 vs. the PCOS group; ♦*p* < .05, ♦♦*p* < .01, the PCOS + SA group vs. the PCOS + Met group. The data are shown as the mean ± SD.

As shown in Figure 10, the p‐Smad2 and p‐Smad3 levels in both the SA and Met treatments were significantly lower than those in the PCOS rats (*p* < .01), and the decrease in the SA‐treated PCOS rats was more pronounced than in the Met‐treated PCOS rats (*p* < .01). Moreover, the Smad4 levels were considerably lower in the SA‐treated and Met‐treated rats than in the PCOS rats (*p* < .01), with a more pronounced decrease in the SA‐treated PCOS rats than in the Met‐treated PCOS rats (*p* < .05). In PCOS rats, the Smad7 levels were significantly lower than those in normal rats (*p* < .01). In the SA‐treated and Met‐treated PCOS groups, the levels of Smad7 proteins were notably higher than those in the PCOS group (*p* < .01 and *p* < .05, respectively), with a more marked increase observed in the SA‐treated PCOS rats than in the Met‐treated PCOS rats (*p* < .01).

### 3.13 Effects of SA on the levels of fibrosis‐related proteins in the ovarian tissues of the PCOS rats

**FIGURE 11:**
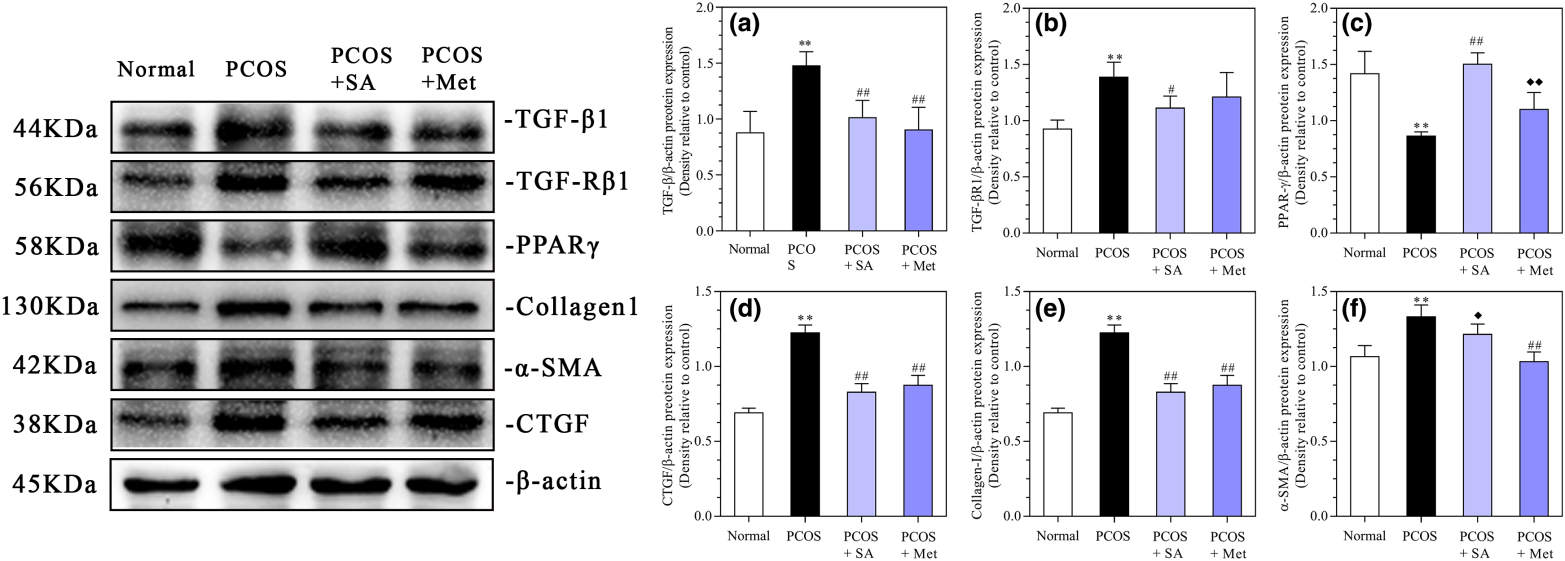
Effects of SA on the protein levels of TGF‐β1 (a), TGF‐βR1 (b), PPAR‐γ (c), collagen I (d), α‐SMA (e), and CTGF (f) in rats with letrozole‐induced PCOS. *p < .05, **p < .01 vs. the normal group; #*p* < .05, ##*p* < .01 vs. the PCOS group; ♦*p* < .05, ♦♦*p* < .01, the PCOS + SA group vs. the PCOS + Met group. The data are shown as the mean ± SD.

As shown in Figure 11, both SA and Met treatments were found to be significantly effective in reducing the levels of TGF‐β1, CTGF, and collagen I in comparison to the PCOS rats (*p* < .01). The TGF‐βR1 and α‐SMA levels of the Met‐ and SA‐treated groups were lower than those of the PCOS rats (*p* < .01 and *p* < .05, respectively). We also observed that the PPAR‐γ levels were increased in both SA‐ and Met‐treated PCOS rats than in the PCOS rats (*p* < .05 and *p* < .01, respectively).

### 3.14 Effect of SA on the ovaries of PCOS rats as assessed by immunohistochemical staining

**FIGURE 12:**
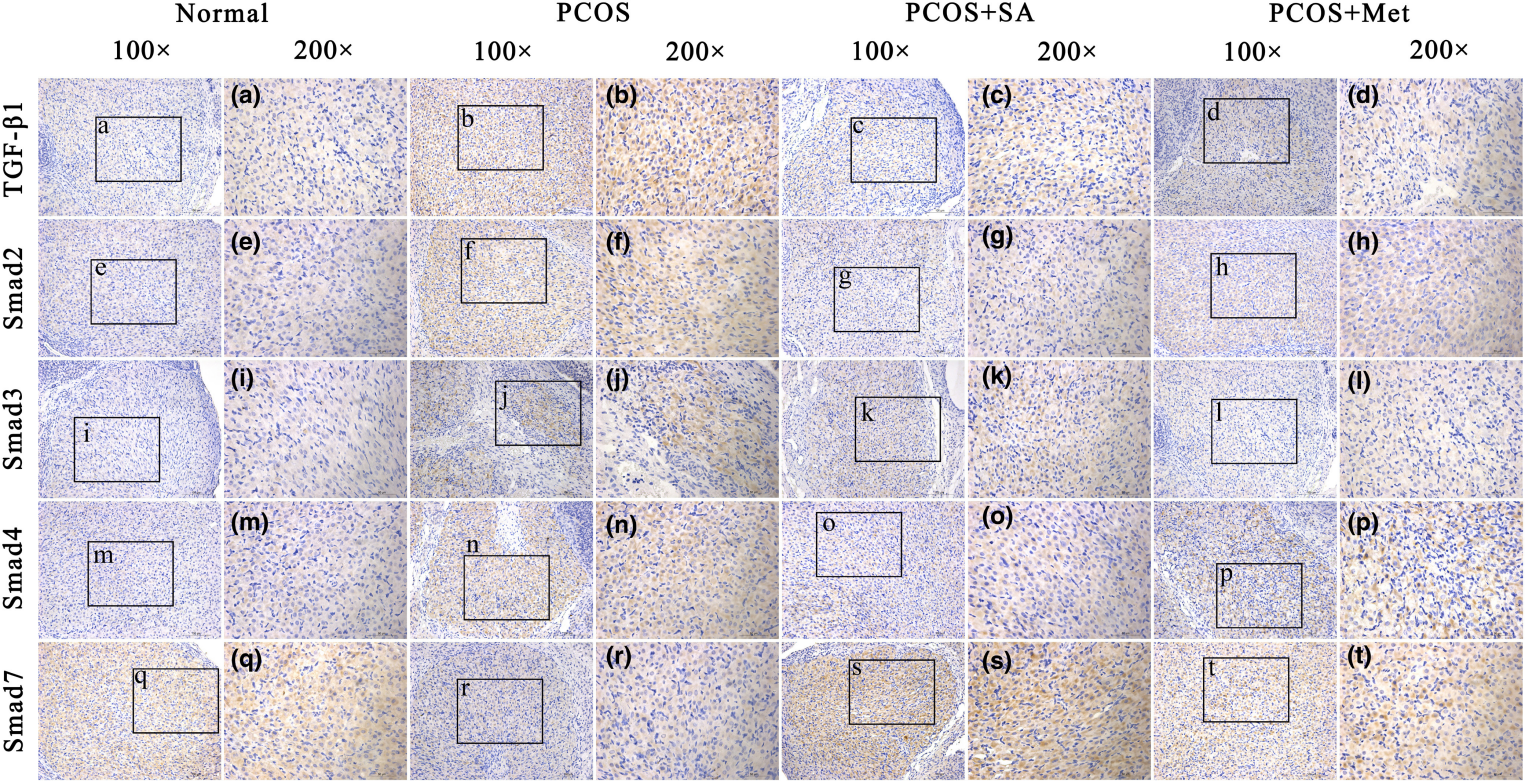
Effects of SA on the protein expression of TGF‐β1, Smad2, Smad3, Smad4, and Smad7 in the ovaries of rats with letrozole‐induced PCOS. Representative immunohistochemistry images of TGF‐β1, Smad2, Smad3, Smad4, and Smad7 protein expression in the ovaries are shown with quantification (scale bar = 100 μm; the black box defines the area to be amplified; high‐magnification scale bar = 50 μm).

The positive staining of TGF‐β1 and Smads was markedly decreased and the positive staining of Smad7 expression was significantly increased in SA‐ or Met‐treated PCOS rats compared with that in PCOS rats (Figure 12). The PCOS + Met group showed more Smad7 reactivity than the PCOS + SA group, with significantly lower levels of TGF‐β1, Smad2, Smad3, and Smad4 detected in the pixel‐based intensities.

## 4 DISCUSSION

Recently, many studies have reported androgens, estrogens, aromatase inhibitors, antiprogestins, surgery, and changes in lifestyle as recommended therapies for PCOS patient's clinical treatment (Furat Rencber et al., 2018). Our investigation of an in vivo rat model was necessary to further elucidate the mechanisms of PCOS and to explore effective treatment methods. Previous reports have suggested that the letrozole‐induced PCOS rat model is a frequently used testing animal model that recapitulates human PCOS in many ways (Reddy et al., 2016; Wang, Yin, & Xu, 2020).

Notably, letrozole administration increases the serum levels of sex hormones (T, LH) and disrupts the LH/FSH ratio balance. Moreover, changes in estrogen‐dependent organ mass, such as a decrease in uterine mass and an increase in ovarian mass, and an increase in body mass gain have been observed, signifying that the rats are overexpressing androgens (Zych et al., 2018). It is generally accepted that PCOS is characterized by increased testosterone levels, and research has shown that this increase in T can have an effect on metabolism and eating habits in women, which could lead to metabolic disorder and weight gain (Barber et al., 2006; Sanchez‐Garrido & Tena‐Sempere, 2020).

Androgens are not the only factor linked to PCOS, as IR has also been associated with the condition. In vitro experiments have revealed that the hypothalamus and pituitary gland contain insulin receptors, which can induce the release of FSH and LH when exposed to increased insulin concentrations (Adeyanju et al., 2019). The hypothalamic–pituitary–gonadal (HPG) axis is the main regulator of reproductive activities. The hormones of rats with letrozole‐induced PCOS exist in a hyperandrogenized state that is responsible for abnormal ovarian physiology (Jahan et al., 2016). Disruption of the usual HPG axis elevates both T and LH. This change is due to excess T feedback to the pituitary gland, which results in excess LH and decreased FSH levels. We suggest that SA may also regulate ovulation by acting on the HPG axis, since T, LH, and LH/FSH levels were significantly lower in the SA treatment group than in the PCOS group. Our findings demonstrate the advantageous effect of SA on hormonal parameters.

Diabetes mellitus has been known to be connected with the emergence and development of PCOS (Joham et al., 2014). Initially, PCOS presents as hyperglycemia, which over time progresses to IR (Boudreaux et al., 2006). Our research revealed that FBG, AUC of OGTT, and HOMA‐IR levels, which are indicative of IR, were significantly increased in PCOS rats. This is consistent with prior studies that showed that letrozole‐treated PCOS rats developed IR (Wang, Yin, & Xu, 2020). Decreases in FBG and the AUC of OGTT were observed in the SA group, indicating the development of normal glucose tolerance. As in previous studies, the SA group may have experienced improved insulin‐mediated glucose uptake with a resultant normal glucose tolerance (Cherng et al., 2013). Hyperinsulinemia and hyperandrogenemia associated with IR may increase lipogenesis and ultimately lead to dyslipidemia (Sam & Dunaif, 2003). Letrozole treatment induced typical disturbances in lipid metabolism in the PCOS group, which is consistent with the literature (Morrone Mda et al., 2016; Sivasinprasasn et al., 2015). Oral administration of SA significantly prevented the increases in the serum levels of TC, TG, and LDL‐C. Our observation is in accord with that of SA ameliorated dyslipidemia by increasing the levels of HDL‐C and decreasing the serum levels of TG in HFD‐fed rats and Syrian hamsters, respectively (Wang et al., 2022; Yang et al., 2019). These findings indicate that SA has a positive impact by improving IR and preventing dyslipidemia.

It has been established that the termination of folliculogenesis is the cause of atresia in ovarian follicles (Lerchbaum & Obermayer‐Pietsch, 2012). We also observed that letrozole induced significant changes in the amounts of AF and CF and decreased the number of CLs in PCOS rats in this study. Hong et al. reported a high number of AFs and CFs with a thin layer of GCs in the ovaries, which is consistent with our finding (Hong et al., 2019). High concentrations of LH in PCOS patients can cause luteinization to occur prematurely, thus stalling the progression of folliculogenesis at the small antral follicle stage (Carvalho et al., 2018).

Administration of letrozole induced an increase in T, which may impair follicle development and increase atresia in PCOS rats (Celik et al., 2018; Kuyucu et al., 2020). However, administration of SA was able to reduce the formation of numerous AFs and CFs in PCOS rats. Transmission electron microscopy (TEM) observations suggested the occurrence of structural changes, such as irregularity of the ZP, disjunction of GCs, attenuation of the GCL, and thickening of the TCL in the CF of PCOS rats. Additionally, degenerative changes in mitochondria, an expanded SER and GER, and many lipid droplets in the cytoplasm of interstitial cells were observed in PCOS rats. Studies on the effects of androgens on follicles have revealed that after androgen treatment, ovarian GCs contain a considerable amount of lipid droplets, large antral follicles with a thin GCL, and a disruption of the basement membrane and GCs (Okutsu et al., 2010; Tessaro et al., 2015). We observed that SA treatment improved the ultrastructure of the ovaries in PCOS rats. Similarly, resveratrol has been found to ameliorate mitochondrial damage and SER and GER expansion in TCs and to improve the ZP, GCL, and TCL (Furat Rencber et al., 2018). The reversal in ovarian structural changes may be a direct consequence of improved serum levels of sex hormones (Celik et al., 2018).

Oxidative stress is considered one of the risk factors for PCOS (Khodaeifar et al., 2018; Zhou et al., 2023). It has been reported that PCOS patients have decreasing levels of glutathione peroxidase (GPx) and SOD (Furat Rencber et al., 2018). The heightened levels of oxidants may modify the environment of the ovaries, leading to augmented androgen production and polycystic ovaries (Sabuncu et al., 2001). Our research revealed that the levels of MDA were increased and the levels of endogenous antioxidant enzymes were decreased in the ovaries of PCOS rats (Figure 9c). The results indicated that SA was capable of decreasing MDA production and augmenting the serum activity of endogenous antioxidants, which showed a capability to shield ovarian tissue from oxidative damage in PCOS rats. Quercetin has been indicated to have an activity that increases the activity of CAT, GSH, SOD, and GPx in PCOS rats (Sarwat Jahan et al., 2018).

In addition, when oxidative stress occurs, pro‐inflammatory cytokines, such as TGF‐β1 and nuclear factor‐kappa B (NF‐κB), are secreted at the injured site. In the short term, inflammation is thought to be beneficial, eliminating pathogens. However, chronic inflammation is harmful and is strongly associated with fibrosis (Luedde & Schwabe, 2011). When NF‐κB signaling is activated in damaged cells, it can activate the expression of genes encoding tumor necrosis factor alpha (TNF‐α) and interleukin 6 (IL‐6), two important pro‐inflammatory cytokines (Qu et al., 2021). The positive feedback loop between NF‐κB and TNF‐α appears to increase levels of inflammation and damage in cells. Therefore, downregulating the activity of related cytokines can inhibit fibrosis (Yang et al., 2021).

The presence of ovarian dysfunction and ovarian fibrosis in PCOS patients makes it challenging for them to conceive. The development of follicles is strongly linked to TGF‐β1 (Omwandho et al., 2010; Young et al., 2017). Overexpression of TGF‐β1 has been observed in PCOS patients (Takahashi et al., 2017), and the related activation of TGF‐β1/Smads also promotes fibrosis (Miao et al., 2012; Zhou et al., 2021). Connective tissue growth factor (CTGF) is a fibrotic factor that encourages the growth of fibroblasts and promotes the formation of the ECM (Wang et al., 2018). After SA intervention, the ovarian protein levels of CTGF and TGF‐βR1 were effectively decreased, while the level of Smad7 was increased. Raish et al. reported that SA inhibited the activation of TGF‐β and could mitigate lung fibrosis in SD rats (Raish et al., 2018). As an integral member of the nuclear hormone receptor family, evidence suggests that PPAR‐γ is a regulator of glucose levels, cellular differentiation, and inflammation (Giampietro et al., 2019; Huang et al., 2019; Soliman et al., 2019). We noted a considerable rise in ovarian PPAR‐γ expression in rats with PCOS following treatment with SA. It has been reported that an elevated amount of PPAR‐γ may suppress collagen I production that is activated by TGF‐β1, implying that these proteins could be integral to organ fibrosis (Zych et al., 2019). The findings from our experiments indicate that SA has the ability to deactivate the TGF‐β1/Smads pathway and enhance the activation of PPARγ. This suggests that SA might have potential therapeutic benefits in preventing fibrosis in the ovary.

## 5 CONCLUSIONS

To summarize, the current study indicates that SA has the ability to regulate metabolic disturbances, improve oxidative stress, and reduce ovarian fibrosis in rats with PCOS. This study illustrates how SA intervention can influence the dysfunction of the TGF‐β1/Smads pathway in PCOS rats. The data of this study revealed that regarding the majority of the findings, there were no significant differences between SA and Met and that SA was superior in ameliorating the PCOS, which indicates that SA may be an alternative to Met in PCOS treatment. Since patients suffer from gastrointestinal symptoms due to the use of metformin, the next step of this study is to establish dose‐dependent experiments in which SA dose is stabilized. Also, other molecules related to this pathway could be investigated with further and more accurate techniques. Thus, the effectiveness of SA can be evaluated precisely. Further controlled clinical studies are required to determine the effects of SA in the treatment of PCOS.

## AUTHOR CONTRIBUTIONS

**Huan Lan:** Investigation (equal); writing – original draft (equal); writing – review and editing (equal). **Zhe‐Wen Dong:** Investigation (equal); writing – original draft (equal); writing – review and editing (equal). **Ming‐Yu Zhang:** Investigation (equal); writing – original draft (equal); writing – review and editing (equal). **Wan‐Ying Li:** Data curation (equal); validation (equal). **Chao‐Jie Chong:** Data curation (equal); validation (equal). **Ya‐Qi Wu:** Data curation (equal); validation (equal). **Zi‐Xian Wang:** Data curation (equal); formal analysis (equal); visualization (equal). **Jun‐Yang Liu:** Data curation (equal); formal analysis (equal); visualization (equal). **Zhi‐Qiang Liu:** Visualization (equal). **Xiao‐Hui Qin:** Visualization (equal). **Tie‐Min Jiang:** Supervision (equal). **Jia‐Le Song:** Funding acquisition (lead); methodology (lead); project administration (lead); resources (lead); supervision (equal); writing – review and editing (equal).

## FUNDING INFORMATION

The present investigation was supported by the Special Funds for Guiding Local Scientific and Technological Development by the Central Government (Grant No. Guike ZY22096025 to J.‐L. S. and T.‐M. J.), National Natural Science Foundation of China (Grant Nos. 81560530, 81760589, 81960590, and 82273630 to J.‐L. S.), the Science and Technology Projects of Guilin (Grant No. 20220139‐8‐2 to J.‐L. S.), the Visiting Scholar Program for Western Light of the Ministry of Education (Grant No. 2023 to J.‐L. S.), the Bagui Outstanding Young Scholars for Guangxi (Grant No. 2023 to J‐.L. S.) the Funding Scheme for High‐Level Overseas Chinese Students' Return of Ministry of Human Resources and Social Security (Grant No. Renshetinghan[2019]160 to J.‐L. S.), the Guangxi Medical and Health Key Cultivation Discipline Construction Project (Grant No. Guiweikejiaofa[2022]4 to J.‐L. S.), and the Opening Project of Guangxi Key Laboratory of Health Care Food Science and Technology, China (Grant No. GXKYSYS202202 to J.‐L. S.).

## CONFLICT OF INTEREST STATEMENT

The authors declare that they have no conflicts of interest.

## Data Availability

The datasets used and/or analyzed during the current study available from the corresponding author on reasonable request.
